# Doxycycline: new tricks for an old drug

**DOI:** 10.18632/oncotarget.5111

**Published:** 2015-08-06

**Authors:** David A. Barbie, Brian K. Kennedy

**Affiliations:** Department of Medical Oncology, Dana-Farber Cancer Institute, Harvard Medical School, Boston; and Broad Institute of MIT and Harvard, Cambridge, MA, USA

Since their original discovery in 1948, tetracycline antibiotics have had a major impact on human health and molecular biology [1]. Beyond their efficacy against a variety of infectious diseases, insights into resistance and discovery of the tetracycline repressor system has yielded an invaluable tool for the inducible control of gene expression. Though originally characterized as selective inhibitors of the bacterial 30S ribosomal subunit [2], tetracyclines appear also to have alternate molecular targets in human tissues and are effective in treating certain skin disorders such as acne and rosacea, which extends beyond a direct antimicrobial effect [3]. Three separate recently published reports characterize “these off-target” activities in detail, suggesting that doxycycline might be repurposed as an anti-cancer therapeutic.

Pulvino et al. utilized the Connectivity Map [4] to search for compounds that reversed an NF-κB signature in HL60 cells, identifying multiple tetracycline family members, including doxycycline [5]. Consistent with this observation, when they treated several different diffuse large B cell lymphoma (DLBCL) cell lines with doxycycline, the authors observed inhibition of NF-κB signaling coupled to decreased cell proliferation and survival. Doxycycline also perturbed STAT3 and ERK activation and reduced the levels of multiple different HSP90 client proteins in these cells, leading the authors to explore its effects on HSP90 activity. Interestingly, doxycycline treatment increased HSP90 ubiquitination and degradation, and more generally increased proteasome-dependent protein neddylation. Through a series of elegant studies, they then determined that these effects were mediated through direct inhibition of the JAMM family metalloproteinase CSN5. Notably, doxycycline was also effective at inhibiting the growth of DLBCL xenografts at physiologically achievable doses, which has prompted the translation of these findings into a clinical trial of single agent doxycycline in relapsed/refractory non-Hodgkins lymphoma patients.

Two other studies characterize a distinct but related role for doxycycline in inhibiting MCF7 breast cancer cell mammosphere formation [6, 7]. De Luca et al. determined that MCF7 mammospheres are particularly sensitive to perturbation of mitochondrial function [7]. Consistent with this observation, over-expression of the transcription factor FOXM1, which promotes stem-like phenotypes, increased mammosphere formation together with upregulation of multiple mitochondrial proteins. Since recent work from this group also demonstrated that doxycycline could suppress tumor-sphere formation by targeting mitochondrial ribosomes, they tested the capacity of doxycycline to reverse FOXM1 driven spherogenesis. Doxycycline indeed prevented the capacity of FOXM1 to promote mammospheres, albeit at very high concentrations. Lamb et al. further determined that doxycycline treatment can inhibit mammosphere formation in primary breast cancer samples, and performed proteomics analysis of MCF7 cells to determine putative doxycycline targets. They noted marked downregulation of DNA-PK by doxycycline treatment, and determined that shRNA-mediated DNA-PK suppression or direct pharmacologic inhibition phenocopied doxycycline, disrupting MCF7 mammosphere formation. Since DNA-PK is involved in radio-resistance, they also evaluated whether doxycycline treatment could synergize with radiation to inhibit MCF7 mammospheres, and observed sensitivity of this subpopulation relative to cells in a monolayer. Finally and similar to Pulvino et al. [5], using a series of reporters, Lamb et al. found that doxycycline treatment of MCF7 cells impairs multiple other signaling pathways, including STAT3 and NRF1/2 [6].

Taken together, these studies suggest that doxycycline, a drug that has been utilized for over 50 years as an antimicrobial agent and more recently in dermatological conditions, could also have efficacy in certain cancers. Despite this promise, several barriers remain to effective translation of these findings to the clinic. First, while Pulvino et al. carefully determined that the doxycycline concentrations used in their experiments match clinical exposure [5], this was not fully explored in the breast cancer studies [5, 7]. Second, a discrete readout of effective target inhibition by doxycycline in human tumors will be necessary to understand whether the doses utilized in clinical trials are indeed high enough to yield prolonged suppression of the tumorigenic pathways. For example, despite equally strong evidence of antitumor efficacy of the antimalarial drug hydroxychloroquine in preclinical studies, treatment of pancreatic adenocarcinoma patients was ineffective and associated with inconsistent autophagy inhibition in peripheral blood cells [8]. Perhaps most importantly, single agent therapy, especially in relapsed aggressive cancers, is less likely to be effective even with proven target inhibition. Thus, negative findings in the lymphoma study, for example, do not necessarily invalidate doxycycline as a useful agent, but may suggest the need to develop combinatorial approaches with more targeted BTK inhibitors, for example. Finally, the characterization of CSN5 and DNA-PK as putative doxycycline targets that mediate its anticancer activities supports the development of more potent and selective inhibitors of these enzymes, which, depending on their therapeutic window, could have an even greater impact for cancer patients.

**Figure 1:**
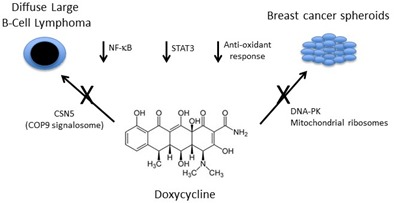
Novel cellular activities for doxycycline relevant to cancer treatment strategies
